# Efficacy and safety of abdominal acupuncture for insomnia: A protocol of systematic review and meta-analysis

**DOI:** 10.1097/MD.0000000000027765

**Published:** 2021-11-19

**Authors:** Zhitao Feng, Zhihong Wang, Zhanshuang Qiu, Tie Li, Lili Zhang, Jiajia Wang, Dashi Ying

**Affiliations:** a Changchun University of traditional Chinese Medicine, College of Acupuncture and Tuina, Changchun University of Traditional Chinese Medicine, No. 1035 Boshuo Road, Changchun, Jilin 131000, P.R. China; b Songyuan Central Hospital, Songyuan 138000, P.R. China; c Jilin Agricultural Science and Technology University, Jilin 132101, China, 3. Changchun Yingshi Medicine Clinics, Changchun 130022, China.

**Keywords:** abdominal acupuncture, insomnia, meta-analysis, protocol, systematic review

## Abstract

**Background:**

Insomnia is characterized by high incidence, easy recurrence, and difficulty in curing. Serious insomnia not only seriously affects the body organ function, but psychological patients also cause great damage. Abdominal acupuncture (AA) has fewer side effects and is increasingly used to treat insomnia. This study aimed to systematically review the effectiveness and safety of abdominal acupuncture in the treatment of insomnia.

**Methods:**

Literature on abdominal acupuncture for insomnia in the PubMed, Excerpt Medica Database(Embase), Cochrane Central Register of Controlled Trials, Web of Science, China National Knowledge Infrastructure Database, China Biomedical Literature Database, Chinese Scientific Journal Database, and Wan Fang databases were searched from the creation of these databases to October 3, 2021. In addition, the reference lists of studies meeting the inclusion criteria will also be searched to achieve a comprehensive retrieval of the maximum. All randomized controlled trials of AA for treating insomnia were included. Two reviewers will conduct literature screening, data extraction, and quality evaluation respectively. The main outcome was the Pittsburgh Sleep Quality Index, and the secondary outcomes included clinical efficacy and safety. RevMan 5.4.1 software was used for mate analysis.

**Results:**

This study aimed to evaluate the current status of AA treatment for insomnia, with the aim of illustrating the effectiveness and safety of abdominal acupuncture.

**Conclusion:**

This study will provide high-quality evidence to evaluate the effectiveness and safety of AA in treating insomnia.

Registration: INPLASY2021100088

## 1 Introduction

### 1.1 Description of the condition

Insomnia is a subjective symptom of poor sleep quality and low sleep duration due to personal reasons such as inability to fall asleep or easy to wake up and difficulty in falling asleep.^[1]^ About 30% to 35% of the world's population suffers from some degree of sleep disorder,^[2]^ and according to an epidemiological survey,^[3]^ about 45.5% of the population in China have different levels of sleep problems, to continue to sleep time is too short, wake up, and difficulty falling asleep as the main symptoms, which is about 50% of the patients showing symptoms of 2 or more at the same time, which seriously influences the patient's emotional and social functions and quality of life. A longitudinal study found that individuals who reported severe insomnia symptoms had a remission rate of only 56% over 10 years.^[4]^ Long-term sleep deprivation can lead to daytime dysfunction, inability to recover physical strength, low work efficiency, poor academic performance, and severe insomnia can increase the risk of coronary heart disease, acute coronary syndrome, anxiety, depression, and other diseases.^[5,6]^ At present, Western medicine is the mainstay of treatment for insomnia, and Western drugs mainly include benzodiazepines, non-benzodiazepines, and melatonin etc. Benzodiazepines have obvious side effects, including drug resistance and rebound insomnia, and long-term use of elderly patients increases the risk of dementia;^[7]^ Non-benzodiazepinescan cause taste disorders, headache, dizziness, and other discomfort,^[8]^ and adverse reactions such as dizziness, taste disturbance, and drowsiness.^[9]^ Based on the side effects of the above drug therapy medical practitioners are forced to explore better complementary and alternative therapies.^[10]^

### 1.2 Description of the intervention

Acupuncture is a treatment that promotes health by inserting needles into specific acupoints on the human body at an appropriate depth. Acupuncture treatment of insomnia has the characteristics of simple operation, nontoxic side effects, positive curative effect, and high safety.^[11]^ Abdominal acupuncture (AA) is an unusual acupuncture therapy that treats systemic disorders by acupuncturing the viscera and meridians in the abdomen.^[12]^ AA therapy is used to treat systemic diseases by adjusting the viscera/meridians (including innate and acquired meridians) through acupuncture at abdominal acupoints to treat systemic diseases. The theory of AA is distinctly different from traditional acupuncture.^[13]^ Some scholars think^[14]^ that acupuncture abdomen with CV8 gas as the core of the abdomen is a regulatory system that CV8 senior body meridian system (innate meridian system), the body's innate meridian system can be divided into shallow deep multilevel three-dimensional structure of the control system, in which the abdomen holographic system is located in the abdominal wall of shallow, traditional meridian system located in the middle of the abdominal wall, abdominal CV8 meridian system located in the deep layer of the wall, this system has been formed in the fetus, and has a strong function of regulating the circulation of qi and blood and the macro-control effect on the whole body. At present, AA is widely used in the treatment of internal, external, women, children, facial diseases, and other diseases and subhealth conditioning, the range of treatment of hundreds of diseases.^[15]^

### 1.3 How the intervention might work

Traditional Chinese Medicine holds the view^[16,17]^ that the underlying pathogenesis for insomnia is an imbalance of Yin and Yang, and it believes that the disease location is mainly in the heart, closely related to the liver, spleen and kidney, gastrointestinal dysfunction leads to poor sleep quality. The abdomen is the main area where Zang-fu organs and their acupoints are distributed, and the 12 meridians are closely related to the abdomen.^[18]^ AA is used to treat insomnia by acupuncture points CV4, CV6, CV8, CV10, CV12, ST24, ST25, ST26, and other special points in the abdomen to stimulate the meridian qi and blood, promote the flow of qi and blood, and adjust the balance of yin and yang in the corresponding internal organs.^[19]^ Western medicine^[20]^ believes that it is the brain-gut axis, which is the theoretical basis of AA for the treatment of insomnia. The brain-gut axis refers to a bidirectional regulatory system of interactions between the gastrointestinal tract and the brain.^[21]^ AA stimulates the abdominal tract and improves sleep quality by regulating the secretion of sleep-related brain-gut peptide content through the brain-gut axis.^[20]^

### 1.4 Why it is important to do this review

AA treatment of insomnia has the characteristics of simple operation, nontoxic side effects, and positive curative effect, and high safety, and AA has now been extensively applied to the clinical treatment of insomnia in china. However, no study has systematically evaluated the safety and efficacy of AA in the treatment of insomnia in a systematic way. The aim of this study was to systematically evaluate the effectiveness and safety of AA for insomnia so that reliable clinical evidence can be provided.

### 1.5 Objectives

To systematically evaluate the effectiveness and safety of AA for insomnia.

## 2 Methods

### 2.1 Study registration

The protocol was registered on INPLASY website (https://inplasy.com/inplasy-2021-10-0088/) and its registration number was INPLASY 2021100088, we will complete this protocol according to the Preferred Reporting Items for Systematic reviews and Meta-Analysis Protocols.^[22]^ The changes are described in our full review, if needed.

### 2.2 Inclusion criteria for study selection

#### 2.2.1 Types of studies

We will only need randomized clinical trials (RCTs) on AA for insomnia, regardless of whether the blind method and allocation concealment are used.

#### 2.2.2 Types of participants

Participants diagnosed with insomnia were included. Diagnosis criteria include the Diagnostic and Statistical Manual of Mental Disorders,^[23,24]^ International Classification of Sleep Disorders,^[25]^ International Statistical Classification of Diseases and Health-Related Problems,^[26]^ and Chinese Classification of Mental Disorders.^[27]^ No restrictions will be applied to age, gender, ethnicity, or source of cases.

#### 2.2.3 Types of interventions and comparisons

The experimental group was treated with abdominal acupuncture. The control group was treated with any therapies, such as Western medicine, acupuncture, moxibustion, auricular needle, and other conventional therapies. The following comparisons were made.

1. AA with Chinese herbal medicine;
2. AA compared with western medicine;
3. AA compared with placebo treatment;
4. AA compared with acupuncture alone;
5. AA compared with moxibustion alone.

If the 2 groups received the same additional active therapy on the basis of the control treatment, the study can also be included.

#### 2.2.4 Types of outcome measures

##### 2.2.4.1 Primary outcomes

Sleep quality will be evaluated using the Pittsburgh Sleep Quality Index ^[28]^ as the primary outcome. Improvement in insomnia can be measured by the clinical efficacy and clinical cure rate. Outcomes can be measured simply at the end of treatment.

Possible primary outcomes included the following:

1. Improvement in overall symptoms of insomnia
2. Improvement in quality of life
3. Clinical efficacy or clinical cure rate

##### 2.2.4.2 Secondary outcomes

Secondary outcomes will include: the total scores of the Insomnia Severity Index;^[29]^ syndrome according to standards for assessing traditional Chinese medicine;^[30]^ and adverse events, such as nausea, dizziness, vomiting, and fatigue.

### 2.3 Search methods for identification of studies

#### 2.3.1 Electronic searches

Two independent reviewers (QZS and WJJ) will search the following 8 databases from the inception to October 2021; including China National Knowledge Infrastructure Database, VIP database, CBM, Wan Fang Data Chinese Database, PubMed, Cochrane Central Register of Controlled Trials, Web of Science, Embase. The combined method of MeSH term and free words was used for literature retrieval. There were no restrictions on language or publication status. The search strategy for PubMed is shown in Table 1. The search strategies of the other databases were established similarly.

**Table 1:** The search strategy for PubMed database.

Number	Search terms
#1	insomnia [MeSH terms]
#2	sleeplessness [MeSH terms]
#3	sleep disorder [MeSH terms]
#4	dyssomnia [MeSH terms]
#5	#1 OR #2 OR #3 OR #4
#6	insomnia[Title/abstract]
#7	sleeplessness[Title/abstract]
#8	sleep disorder [Title/abstract]
#9	dyssomnia [Title/abstract]
#10	#6 OR #7 OR #8 OR #9
#11	#5 OR #10
#12	abdominal acupuncture [MeSH terms]
#13	abdominal needle [MeSH terms]
#14	abdominal Electroacupuncture [MeSH terms]
#15	Abdomen acupuncture[MeSH terms]
#16	Abdomen needle[MeSH terms]
#17	Abdomen Electroacupuncture[MeSH terms]
#18	#12 OR #13 OR #14 OR #15 OR #16 OR #17
#19	abdominal acupuncture [Title/abstract]
#20	abdominal needle [Title/abstract]
#21	abdominal Electroacupuncture [Title/abstract]
#22	Abdomen acupuncture [Title/abstract]
#23	Abdomen needle [Title/abstract]
#24	Abdomen Electroacupuncture [Title/abstract]
#25	#19 OR #20 OR #21OR#22OR#23 OR#24
#26	#18OR#25
#27	Clinical trail [MeSH terms]
#28	Randomized clinical trail[MeSH terms]
#29	Randomized controlled trail [MeSH terms]
#30	#27OR #28OR #29
#31	Randomized clinical trail [Title/abstract]
#32	Randomized controlled trail[Title/abstract]
#33	RCT [Title/abstract]
#34	Clinical trail [Title/abstract]
#35	Random∗[Title/abstract]
#36	Clinical trail [Publication Type]
#37	#31 OR #32 OR #33 OR #34 OR #35 OR #36
#38	#30 OR #37
#39	#11 AND #26 AND #38

#### 2.3.2 Searching other resources

To augment the results of the database search, the bibliographies of the identified studies, relevant reports, and reviews will be manually searched. We will also contact the relevant experts and organizations for information about unpublished and ongoing studies.

### 2.4 Data collection and analysis

#### 2.4.1 Selection of studies

**Figure 1:**
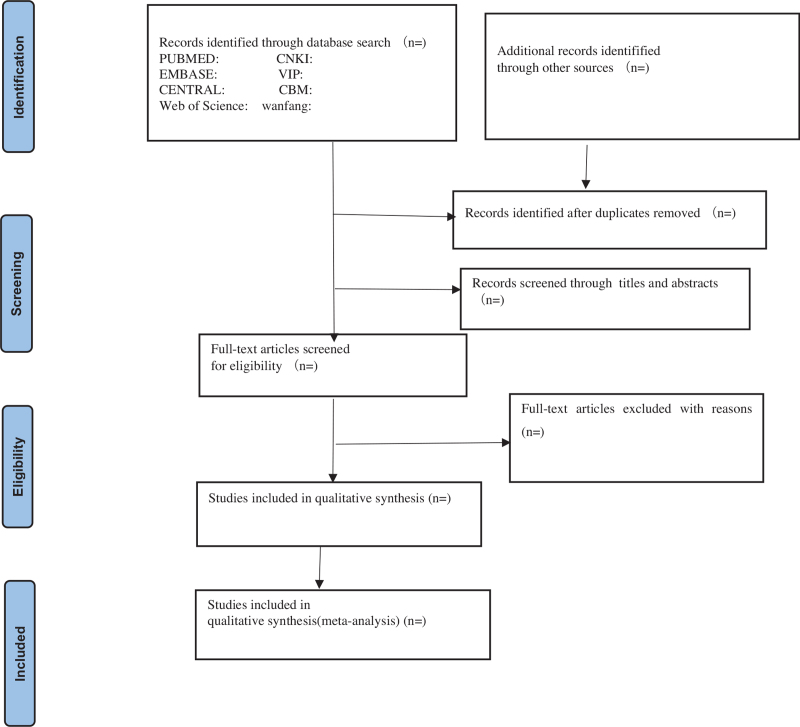
Flow chart of the search process.

We will first use Note Express software (V.3.2) to remove duplicates, and then screen the retrieved studies separately by 2 reviewers (ZLL and QZS) according to the inclusion criteria. Two reviewers (ZLL and QZS) will exclude the papers that do not meet the inclusion criteria by reading the titles and abstracts. Then, the reviewers will check the full texts to determine the final decision according to the criteria. All the screening processes were conducted independently. If the articles information is insufficient, we will try to contact the authors to obtain the necessary details. When 2 reviewers have different opinions, the final decision will be made by a third reviewer (LT). The selection flow process is shown in the PRISMA flow chart (Fig. 1).

#### 2.4.2 Data extraction and management

First, we design an extraction form that meets the purpose of this system review, which will include the following information from the included studies: participant characteristics, interventions, outcomes, and adverse events. Two investigators (QZS and ZLL) will independently complete the data collection form for all eligible studies. The corresponding authors will be contacted to request insufficient or missing information. Disagreements will be resolved by discussion or by appealing to a third author (WZH). The data were stored in Microsoft Excel.

#### 2.4.3 Assessment of risk of bias in included studies

We used the Cochrane risk assessment tool to assess the risk of bias.^[31]^ The methodological quality of RCTs will be independently evaluated by 2 reviewers (FZT and QZS). The following 7 items will be included: random sequence generation, allocation concealment, blinding of participants and caregivers, blinding of outcome evaluator, incomplete outcome data, selective reporting, and other bias. High, low, and unclear assessments were performed for each item. Any disagreement between the 2 reviewers (FZT and QZS) will be resolved by a discussion. Further disagreements were arbitrated by the third author (WZH).

#### 2.4.4 Measures of treatment effect

We will use the mean difference or standard mean difference with 95% confidence intervals as the effect measure for continuous data. Dichotomous outcomes will be analyzed by the risk ratio with 95% confidence intervals.

#### 2.4.5 Dealing with missing data

When there are events in the reports that are unclear or do not report data, we will contact the author by phone or email to obtain complete information.

#### 2.4.6 Assessment of heterogeneity and data synthesis

##### 2.4.6.1 Assessment of heterogeneity

We will use RevMan5.4.1 software to detect the heterogeneity between studies.^[32]^ When *P* < .01, I^2^ > 50%, there is significant heterogeneity between studies; otherwise, heterogeneity is acceptable.

##### 2.4.6.2 Data synthesis

RevMan5.4.1 will be used for all statistical analyses. We used the random effects model to merge the data. The results of the meta-analyses are presented as forest plots. When the results are unsuitable for combination due to clinical or the methodological heterogeneity, we will perform a descriptive analysis.

#### 2.4.7 Sensitivity analysis

If the result shows high heterogeneity (the I^2^ test is > 50%), we will conduct a sensitivity analysis. We will then acquire a stable result of our study.

#### 2.4.8 Subgroup analysis

If there are adequate studies and available data, we will conduct subgroup analysis for different syndrome types of insomnia to explain the heterogeneity among studies.

#### 2.4.9 Assessment of reporting biases

Funnel plots were used to explore the publication bias when 10 or more trials were included.

#### 2.4.10 Grading the quality of evidence

The certainty of a body of evidence will be assessed by using the approach developed by the Grades of Recommendation, Assessment, Development and Evaluation Working Group (GRADE Working Group),^[33]^ involving risk of bias, heterogeneity, indirectness, imprecision, publication bias, and other domains. The certainty level will be rated as high, moderate, low, or very low, and the strength of evidence recommendation will be judged as strong or weak.

### 2.5 Ethics and dissemination

In this study, no individual data from participants were involved, so ethics approval was not required. This systematic review will be published in a peer-reviewed journal.

## 3 Discussion

Insomnia is most common in the elderly, women, mental workers, and people without social backgrounds, and has a serious impact on health and quality of life.^[34]^ Due to obvious side effects of drug treatment for insomnia and the high price of cognitive behavioral therapy for insomnia, heavy economic burden for patients, and high requirements for doctors, it is not widely used in clinical practice at domestic and overseas. AA for insomnia has the advantages of quick effect, safety, low price, definite curative effect, and no side effects. AA is widely used in clinical treatment of insomnia in China. Therefore, it is necessary to conduct a systematic review to establish convincing evidence to prove the effectiveness and safety of AA for insomnia. Due to the uneven quality of the literature, such as few outcome indicators, long publication time, and lack of evidence quality evaluation, the results may be uncertain. Therefore, we will adopt a more rigorous method for systematic review method. We hope that this evidence can help clinicians and health policymakers make clinical decisions on insomnia and provide good news to patients. However, there may be some potential limitations to this systematic evaluation. First, due to the different types of insomnia, heterogeneity may be greater. Second, the quality of RCTs may be low and there is a risk of bias.

## Acknowledgments

This study was supported by funding from the National Key Basic Research Development Program Project 973 project (2014CB543100), the National Natural Science Fund No. 86174092.

All relevant data are within the paper and its Supporting Information files.

## Author contributions

**Conceptualization:** Zhihong Wang, Zhitao Feng.

**Data curation:** Zhanshuang Qiu, LiLi Zhang, Tie Li.

**Funding acquisition:** Zhihong Wang.

**Investigation:** Zhitao Feng, Lili Zhang, Jiajia Wang.

**Methodology:** Zhihong Wang, Tie Li.

**Project administration:** Zhihong Wang, Dashi Ying.

**Software:** Zhitao Feng, Zhanshuang Qiu.

**Supervision:** Zhihong Wang, Tie Li.

**Writing – original draft:** Zhitao Feng, Zhanshuang Qiu.

**Writing – review & editing:** Zhitao Feng, Zhihong Wang, Tie Li.
